# Cellular Self-Digestion and Persistence in Bacteria

**DOI:** 10.3390/microorganisms9112269

**Published:** 2021-10-31

**Authors:** Sayed Golam Mohiuddin, Sreyashi Ghosh, Han G. Ngo, Shayne Sensenbach, Prashant Karki, Narendra K. Dewangan, Vahideh Angardi, Mehmet A. Orman

**Affiliations:** Department of Chemical and Biomolecular Engineering, University of Houston, Houston, TX 77004, USA; smohiuddin3@uh.edu (S.G.M.); sghosh15@uh.edu (S.G.); hannyngo151@gmail.com (H.G.N.); shayne.sensenbach@gmail.com (S.S.); prasubba.karki@gmail.com (P.K.); ndewangan@uh.edu (N.K.D.); vahidehanghardi@gmail.com (V.A.)

**Keywords:** self-digestion, autophagy, bacterial persisters, intracellular degradation, stationary-phase metabolism, protein degradation, RNA degradation, lipid degradation, viable but non-culturable cells

## Abstract

Cellular self-digestion is an evolutionarily conserved process occurring in prokaryotic cells that enables survival under stressful conditions by recycling essential energy molecules. Self-digestion, which is triggered by extracellular stress conditions, such as nutrient depletion and overpopulation, induces degradation of intracellular components. This self-inflicted damage renders the bacterium less fit to produce building blocks and resume growth upon exposure to fresh nutrients. However, self-digestion may also provide temporary protection from antibiotics until the self-digestion-mediated damage is repaired. In fact, many persistence mechanisms identified to date may be directly or indirectly related to self-digestion, as these processes are also mediated by many degradative enzymes, including proteases and ribonucleases (RNases). In this review article, we will discuss the potential roles of self-digestion in bacterial persistence.

## 1. Introduction

Antibiotic failure is a growing concern worldwide [1], and persister cells—a small subpopulation of transiently non-growing drug-tolerant cells within a larger bacterial cell population—significantly contribute to this problem by facilitating the emergence of antibiotic-resistant mutants and the recurrence of microbial infections [2,3,4,5,6]. Because it is not mediated by heritable mutations, the persister state is reversible, and persister formation can occur in response to multiple environmental triggers, including antibiotic treatment [7,8], nutrient depletion [9,10,11], temperature [12], and pH [13,14,15]. A number of pathways have been implicated in persister formation, including the SOS response [7,16,17], the ppGpp-mediated stringent response [10,18], quorum sensing [19,20], and cellular aging [21]. In addition, reactive oxygen species (ROS) [22,23,24], toxin/antitoxin (TA) systems [25,26], and intracellular proteases [15,27] have been involved in this process. Notably, persistence seems to be a conserved phenomenon that has been reported in many cell types, including cancer cells [28,29,30,31]. Persister cells have been identified in almost every pathogenic or nonpathogenic microbial species studied to date, including *Escherichia coli*, *Acinetobacter baumannii*, *Cyanobacteria*, *Salmonella Typhimurium*, *Vibrio cholerae*, *Xylella fastidiosa*, *Staphylococcus aureus*, *Mycobacterium tuberculosis*, *Candida albicans*, and *Saccharomyces cerevisiae* [31]. Although dormancy is thought to be the prevailing trait that makes these persister phenotypes tolerant to external stresses [32,33,34,35,36], a significant number of studies have shown that persister cells are heterogeneous [37,38,39,40,41,42,43,44,45,46] and can escape cell death pathways through a diverse range of epigenetic mechanisms [10,18,19,47,48,49].

From an evolutionary perspective, self-digestion, known as autophagy in eukaryotes, is an important survival mechanism. This complex intracellular degradation is coordinated by many regulatory proteins and checkpoint kinases and has been well documented in mammalian cells [50,51], although rarely studied in bacteria. Autophagic mechanisms are associated with a diverse range of enzymes, including proteases, nucleases, glycosidases, lipases, and phosphatases, which are essential components of the intracellular degradation machineries [52,53]. Although self-digestion temporarily provides energy to cells in a non-nutritive environment or under stress conditions, this process may result in growth arrest or death due to the degradation of intracellular components. Critically, in some cases, these components are targets of conventional antibiotics. Self-digestion can therefore act as a double-edge sword; while excessive intracellular degradation can eventually result in cell death and the elimination of persisters [27,54], moderate degradation might only cause growth arrest and may render persisters transiently resistant to antibiotics [55]. Thus, mapping this complex network that may mediate persister formation will not only enhance our knowledge of persister cell physiology, but also provide novel antipersister therapeutic approaches.

A number of outstanding reviews on bacterial persister formation/reawakening mechanisms, physiology, evolutionary perspectives, and treatment strategies have been published in the literature to date [31,44,56,57,58,59,60,61,62,63,64,65,66]. Therefore, in this review article, we will particularly focus on the potential links between self-digestion and bacterial persistence. Specifically, we will first discuss the underlying reasons for self-digestion in bacteria and why it is an important survival mechanism, while highlighting the potential degradative mechanisms (including for those protein, ribonucleic acid (RNA), and lipids) that may induce persister cell formation. We will also explore how self-digestion may shape persister cell metabolism. Finally, we will briefly discuss autophagy to highlight the evolutionarily conserved aspects of the relationship between intracellular degradation and drug tolerance.

## 2. Why Does Cellular Self-Digestion Occur?

Self-digestion in bacteria is a dynamic process that degrades and removes unnecessary or dysfunctional cellular components within the cytoplasm, allowing cells to perform structured deterioration, while recycling key cellular constituents [67,68,69,70,71,72]. Although intracellular degradation continually occurs within bacteria to maintain cellular hemostasis, self-digestion occurs in response to specific stressors, such as starvation and nutrient deprivation [70,73,74]. In nature, most microorganisms are not afforded an abundance of resources needed for growth and reproduction processes [74], and many bacterial species encounter a scarcity of nutrients in their respective ecosystems [75,76]. This lack of nutrients may force cells to enter a quiescent physiological state [77], such as dormancy, to survive in nutrient-limited conditions. However, even in such states, microorganisms may not necessarily be fully dormant during the entire starvation period [74,78], which in nature, can vary from days to years, depending on the ecosystem [74]. For example, microorganisms in salt mines, deep-ocean habitats, ancient rocks, and caves can face starvation periods that may be as lengthy as thousands of years [74,75,76,78,79,80,81,82,83]. Some bacterial species (e.g., *Acetonema, Bacillus, Clostridium, Heliobacterium*) can survive this prolonged starvation by forming endospores. Sporulation, a tightly regulated, genetically programmed cellular process, is distinct compared to the normal bacterial growth where cells divide by binary fission to generate two identical daughter cells. In contrast, an asymmetric division is observed during sporulation, resulting in generation of two cells within the same cell wall: a small forespore and a large mother cell that engulfs the forespore [84,85,86]. The fully formed spore is released to the environment when the mother cell is completely degraded [84,85,86,87]. We note that persisters and endospores are two distinct phenotypes. While persister cells are often referred to a small subpopulation of non-growing cells in a cell population that can form stochastically or deterministically, endospores are referred to dormant, nonproductive phenotypes produced by certain bacterial species as a result of extreme stress conditions.

Unlike in natural environments, bacteria in the laboratory are provided with ample nutrients to support maximum growth [88]. In such nutrient-rich cultures, the doubling time of some bacteria (e.g., *Clostridium perfringens*) can be as fast as 10 min during exponential growth [89]. Upon exhaustion of nutrients, however, bacteria enter the so-called stationary phase, wherein growth cessation occurs, although the cells still exhibit certain metabolic activities [67,74,90]. Once bacteria transit from exponential phase to stationary phase, they develop various strategies (e.g., self-digestion) to survive in their nutrient-depleted environment [67,90,91,92,93].

Notably, before initiating any sort of survival response, upon entrance to stationary phase, bacteria undergo several morphological changes. In particular, cells in stationary phase become smaller in size, and rod-shaped bacteria become more spherical in shape. This results from changes to the cell membrane and cell wall. For example, in *Escherichia coli* entering stationary phase, the cell envelope becomes more rigid, and stress-bearing peptidoglycan layers increase from 0.7–0.8% to 1.4–1.9% of the cell’s dry weight [94]. Moreover, the cell wall becomes more highly cross-linked, and bacteria experience reduced membrane fluidity [67,95].

Reductive division, a process by which cells complete their final rounds of cell division in early-stationary phase, without increasing their biomass, also causes them to become smaller and to adopt a spherical shape [67,96]. This spherical morphology is mostly governed by the RpoS-dependent BolA protein [97,98], which regulates genes encoding for penicillin-binding protein (PBP)5, PBP6, and class C B-lactamases [98]. Overexpression of BolA drastically decreases outer membrane permeability and induces biofilm formation and persistence [99,100]. Aerobic respiration control protein A (ArcA) is another DNA-binding transcriptional regulator that is induced during stationary phase and involved in reductive division [101]. Deletion of *arcA* results in poor survivability in the absence of exogenous carbon sources [101]. Although, in *E. coli* and *Staphylococcus aureus,* levels of persisters in Δ*arcA* cells are not significantly different than in wild-type (WT) cells [47,102]. Δ*arcA* cells are unable to undergo reductive division and remain longer in stationary phase [101]. After undergoing reductive division, cells encounter a continuous reduction in cell size due to the degradation of endogenous cellular components [67]. Critically, these cells become highly tolerant to stress conditions, and a number of studies have reported that small [55,103] and aging [21,104] cells in stationary-phase cultures display increased antibiotic tolerance, resulting from their extensive morphological and physiological alterations.

## 3. What Are the Global Regulators of the Cellular Self-Digestion Network?

Self-digestion is initiated at the beginning of the starvation response, when bacteria begin to degrade their cytoplasmic membrane, cell wall, proteins, RNA, and DNA [67,105,106,107,108]. This process is mediated by a large number of degradative enzymes found in the bacterial cytoplasm, membrane, and periplasm [109]. Critically, despite their prevalence, the regulatory mechanisms that control expression of these molecules at the transcriptional level are largely unknown. Upon nutrient depletion or when cells enter stationary phase, significant changes in the intracellular levels of many global regulators, including DksA, Rpos, and ppGpp, are observed [90,110,111,112,113,114,115,116,117]. However, these regulators have many functions beyond just protecting the cell during starvation. Consequently, they are also induced in response to various stresses, such as oxidative stress, heat/cold shock, osmotic pressure, low pH, ultraviolet (UV)-induced DNA damage, high cell density, and toxic chemicals [115,118,119,120,121,122,123,124,125].

One such regulator is the stationary-phase transcription factor σ^S^ (i.e., the alternative sigma factor). Expression of this protein, which is encoded by the *rpoS* gene, dramatically increases in stationary phase, where it functions to regulate the expression of numerous stress-related genes [90,112,113,114,115,116,117,126]. Strains lacking σ^S^ show rapid cessation of growth upon introduction to starvation conditions in *E. coli* [127]. Notably, although the formation of bacterial persister cells in response to polyamines has been attributed to overexpression of *rpoS* [128], the effect of *rpoS* deletion on persistence depends on the experimental conditions and strains being used [129,130,131]. However, RpoS is not the only sigma factor in bacteria; other well-studied sigma factors include σ^B^, σ^C^, σ^D^, and σ^H^ in *Bacillus subtilis* [132,133,134,135], σ^E^, σ^H^, and σ^S^ in *Pseudomonas aeruginosa* [136,137], and σ^B^, σ^H^, and σ^M^ in *Corynebacterium glutamicum* [138]. In *E. coli*, sigma factors σ^H^ and σ^N^ are overexpressed during stationary phase and can help cells survive during starvation [139,140]. Similarly, the absence of σ^E^ drastically compromises viability of *Salmonella* cells in stationary phase [141].

In addition to sigma factors, the transcription factor DksA and the alarmone molecule (p)ppGpp, which is synthesized by the RelA and SpoT enzymes, form a global regulator of the stringent response that is activated upon carbon source depletion or amino acid starvation [142,143,144]. Induction of the stringent response has been shown to induce persister formation in bacteria [10,45,145], and numerous research groups have reported reduced persister levels in Δ*relA*, Δ*spoT*, and Δ*dksA* strains [11,18,146,147,148,149]. However, although molecules such as RpoS, DksA, and ppGpp have been extensively studied in the field of persister research [10,11,18,146,147,148,149], the question of whether they directly regulate persister mechanisms associated with self-digestion has yet to be answered. It is also possible that degradative enzymes may be constitutively expressed, which results in the accumulation of the enzymes in stationary-phase cells; however, this hypothesis needs to be verified.

## 4. Intracellular Degradation Mechanisms

During self-digestion, cells may initially restrain themselves from degrading essential components that are needed to help them generate energy for survival [74]. Accumulation of glycogen and poly-β-hydroxybutyric acid during exponential growth further ensures bacterial survival during carbon starvation [95,150,151,152]. Thus, bacteria that cannot gather adequate energy-rich molecules, such as glycogen, may rapidly degrade their major cellular components, including RNAs, proteins, and lipids, to generate energy molecules [150,151,152,153,154,155], and this will be further discussed in more detail below.

### 4.1. RNA Degradation

RNases comprise a group of hydrolytic enzymes that degrade RNA into smaller components [68]. There are two main types of ribonucleases: endoribonucleases (e.g., RNase P, III, BN, HI/II, I, E, G, and LS) and exoribonucleases (e.g., RNase D, T, PH, R and II, and PNPase) [68,156]. Endoribonucleases cleave single-stranded RNAs (ssRNA) or double-stranded RNAs (dsRNA) at internal phosphodiester bonds, whereas exoribonucleases cleave either the 3′ end or 5′ end of an RNA molecule [156,157]. In addition to their ability to degrade RNA, RNases play diverse roles in RNA metabolism, functioning in RNA maturation, quality control, and regulation [68]. RNases from different bacteria are generally conserved; however, some RNases can be species-specific, such as the *B. subtilis* RNase M5 (5S rRNA maturation), which is not present in *E. coli* [158].

Degradation of stable RNAs via the action of RNases occurs in response to depletion of nutrient sources during starvation [159]. As ribosomes account for the majority of RNAs in a cell, the RNAs degraded in this process are primarily ribosomal RNAs (rRNAs). These molecules are plentiful in cells and store substantial amounts of nutrients and energy that can be consumed during starvation [160]. For example, approximately 90% of 23S rRNA and 50% of 16S rRNA are degraded in *Salmonella* strains upon entry into the stationary phase in Luria–Bertani cultures [69]. Transfer RNAs (tRNAs) were found to be more stable in *E. coli* cells during phosphate starvation in a minimal medium [153]. Further, under starvation conditions, more than 70% of rRNA produced remains unused by ribosomes and is degraded in *E. coli* [161], suggesting the presence of a conserved molecular mechanism for rRNA degradation. Cells experiencing starvation from specific nutrients, such as carbon [162], nitrogen [163], phosphate [72], and magnesium (II) [164], may digest their rRNAs at different rates [153,160], although the exact extent of rRNA degradation under starvation conditions remains poorly understood. *E. coli* may digest their ribosomes in a unique manner, and once degradation begins, the 30S ribosomal subunit seems to perish quicker than the 50S subunit [153]. Kaplan and Apirion demonstrated that in starved cells, ribosomal degradation proceeds from polysomes to monosomes to ribosomal subunits [160]. The RNA pieces produced by this process are then further degraded to nucleotides by RNase II and PNPase [160].

Although ribosome dimerization and complex formation with their associated proteins has recently been shown to play a critical role in the resuscitation of rifampicin-induced antibiotic-tolerant cells [63,165,166], a direct correlation between the ability of mutant strains (exhibiting different RNase activities) to recover from starvation and their capacity to degrade RNA has been long established [160]. Specifically, strains that rapidly degrade RNA survive starvation better than more slowly degrading strains [160], suggesting a link between RNase activity and persister formation. RNases associated with type II TA systems, such as MqsR/MqsA [167], MazF/MazE [168], RelE/RelB [169], YoeB/YefM [34], and YafQ/DinJ [170], have been well-studied in the field of persister research. TA systems contain pairs of genes, one of which encodes a stable toxin and another that encodes an unstable antitoxin [171]. Antitoxins, under normal growth conditions, degrade, neutralize, or inhibit the associated toxin molecule [171]. Although the deletion of type II toxin molecules or TA systems, including *chpB, mazF, relB/relE, yefM/yoeB, dinJ/yafQ, higB/higA, prlF/yhaV, yafN/O, mqsR/mqsA,* and *hicA/hicB*, does not affect bacterial persistence [172], it is well established that toxins can induce cell cycle arrest by disrupting various cellular processes [171,173,174]. One of the first TA systems to be associated with persistence was the HipA/HipB system. HipA encodes a kinase that can inactivate synthesis of glutamyl tRNA synthetase [175,176], and one HipA mutant, HipA7, was found to show an approximately 100–1000-fold increase in persister levels [25]. In contrast, deletion of the HipA/HipB TA system results in an ~10–100-fold decrease in persister level [169]. Cho and colleagues further showed that rRNAs and tRNAs are primarily degraded in HipA-mediated persister cells, and ribosomes exist in their inactive forms in these cells [177]. MqsR encodes a ribonuclease that interferes with transcription by cleaving mRNA specifically at GCU sites [178], and the MqsR/MqsA TA system is another example of a case where overexpression or deletion of the TA system leads to either an increase or decrease, respectively, in persister formation [167]. Similarly, the RelE/RelB TA system, which has also been shown to aid in persistence, contains an RNase that cleaves mRNA in ribosomal site A, leading to inhibition of translation and growth arrest [179]. The toxin MazF, which is located downstream of the *relE* gene, also cleaves mRNAs at an ACA sequence at the 5′ end [180]. Although the biological role of MazF/MazE remains a subject of debate, studies have shown it plays a significant role in programmed cell death [181]. A recent study by Harrison et al. further showed that deletion of *YafQ* from the YafQ/DinJ TA system results in an approximately 2400-fold decrease in cell survival in antibiotic-exposed biofilms [170]. Collectively, these results support a key role for TA systems in bacterial persistence, although recent controversies [172,182,183] indicate that more studies are needed to fully elucidate the connection between TA system and the persistence state.

### 4.2. Protein Degradation

Proteases play a vital role in maintaining basal levels of regulatory proteins and removing misfolded and abnormal proteins from bacteria. These proteolytic enzymes can be divided into two groups, based on whether the cleavage position is inside the protein (endopeptidases or proteinases) or at the terminus (exopeptidases or peptidases) [184]. Depending on their cellular location, proteases can also be classified as cytoplasmic (e.g., Lon, ClpAP, ClpXP, HslUV), periplasmic (e.g., Tsp, HtrA, protease III), or membrane proteases (e.g., FtsH, OmpT) [71,185,186,187,188,189,190]. Although there are a few examples of energy-independent proteases, including protease III, VII, HtrA, Mi, and Tsp, the majority of intracellular proteolytic processes operate at the post-translational level and are powered by ATP hydrolysis [71,109]. Specifically, ATP hydrolysis is required to change the conformation of the protease, unfold the substrate, and pass the substrate through the protease active site [191].

Energy-dependent proteases are highly significant in *E. coli* and are responsible for more than 90% of the proteolytic activity taking place in the cytoplasm [71]. This model organism encodes five different AAA+ (ATPases associated with diverse cellular activities) proteases, Lon, ClpXP, ClpAP, HslUV, and ClpYQ, as well as the essential protease FtsH [192,193,194]. The ATPase and proteolytic domains of these proteins are located at the bacterial cytoplasm; the former is responsible for initiation of substrate degradation by the ATP-dependent unfoldase and translocation of the unfolded protein to the proteolytic domain. Here, it is further broken down into smaller peptides, five to 25 amino acids in length, with the help of peptidase [193,195].

In some cases, protein degradation can occur through a multistep process, with initial cleavage mediated by an ATP-dependent protease (rate-limiting step), followed by digestion via ATP-independent proteases and peptidases, ultimately leading to formation of free amino acids [71,196]. Peptidases display very high levels of activity, and only a small number of intermediate products of proteolysis are found in cells [71]. Proteases, particularly the ATP-dependent proteases, are extremely substrate-specific due to their structural features. They are also much larger in size (up to 750 kDa) than peptidases active in the extracellular medium, such as trypsin, which has a size of less than 50 kDa [188,196]. From a thermodynamic point of view, protein degradation is spontaneous, even for ATP-dependent proteases such as Lon and ClpAP, which can degrade a trace amount of small peptides without the need of ATP [197,198,199].

Lon, the first and most widely studied ATP-dependent protease, is a cytoplasmic serine protease, which is considered to be the primary protease for quality control in *E. coli* [200]. It is involved in the degradation of misfolded proteins, along with certain major regulatory proteins, such as the cell division regulator, SulA, and the capsule synthesis regulator, RcsA [201,202,203,204,205,206,207]. Lon can play an active role in persister formation, as it degrades several labile antitoxins of type II TA modules, releasing intra-bacterial toxins that cause growth inhibition. For example, the antitoxin RelB is degraded by Lon, decreasing intracellular toxin–antitoxin levels and leading to the accumulation of free RelE toxin, which induces global inhibition of translation [208]. Other antitoxins degraded by Lon include CcdA, HipB, and MazE [208,209,210].

Intriguingly, reduced levels of fluoroquinolone-tolerant persisters were observed in *Lon*-deficient cells [13,211], although the question of whether this phenotype is dependent on the activity of TA modules is highly debatable [13,211,212,213]. As part of the DNA-damage response, the cell division inhibitor, SulA, is upregulated when the cells are treated with fluoroquinolone antibiotics. Thus, in the absence of Lon, SulA accumulation may also affect persister cell survival [13,211,213,214].

Lon might also not be the only protease involved in TA module activation, as researchers have found that Clp proteases are also capable of degrading several antitoxins, including MazE and DinJ [215,216]. The Clp chaperone–protease family is another major group of ATP-dependent serine peptidases that are responsible for the degradation of a huge number of proteins. ClpAP and ClpXP both contain the proteolytic component, ClpP, which along with the co-factor ClpS, has been found to be required for environmental adaptation and extended viability in stationary phase. ClpS adapter protein specifically inhibits the degradation of ssrA-tagged substrates by ClpAP but directs ClpAP to degrade aggregated proteins and possibly N-end rule substrates [217,218]. In addition, the ClpAP protease is responsible for activation of the ParDE TA system by degrading the ParD antitoxin, resulting in transient growth arrest [219]. On the other hand, acyldepsipeptide antibiotic (ADEP4)-activated ClpP can become highly non-specific and kill growth-inhibited persister cells by degradation over 400 intracellular targets [27]. Altogether, despite the fact that both the Lon and Clp proteases have been extensively studied in the field of persister research, it remains unclear whether other ATP-dependent or energy-independent proteases may also function as critical persister molecules.

### 4.3. Lipid Degradation

During the transition to stationary phase, the fatty acid degradation regulon is overexpressed and provides a carbon source to starved cells via the digestion of membrane components [220]. FadR is a global regulator of lipid and fatty acid metabolism and acts as a switch between fatty acid β-oxidation and fatty acid biosynthesis [221]. FadR represses several genes and inhibits transcription of the *fad* genes [221,222], and its activity is modulated by the long-chain acyl-CoA thioester small effector molecule, which binds directly to FadR [221]. This protein complex (FadR-acyl-CoA thioester) cannot bind to the operator sequence in the promoter of the fatty acid degradative genes, leading to *fad* gene activation [221,223].

In response to carbon starvation, derepression of the FadR regulon results in digestion of membrane lipids, yielding fatty acids that are utilized by acyl-CoA synthetase to generate acyl-CoA [224]. The β-oxidation enzymes encoded by *fadBA*, *fadE*, *fadFG*, and *fadH* then convert acyl-CoA into acetyl-CoA, which is an important source of energy during starvation [224]. Consistent with these observations, it has been shown that a strain lacking acetyl-CoA dehydrogenase (encoded by *fadF*) barely survives carbon starvation [224]. Of note, in *E. coli,* lipid degradation occurs on the outer and inner membrane, as there are no intracellular forms of lipid storage in this bacterium [67].

Notably, cells can also activate emergency derepression pathways independently of FadR in stationary phase to survive carbon starvation [225]. Thus, in stationary phase, the *fad* genes become active via activity of ppGpp-programmed RNA polymerase together with cyclic adenosine monophosphate (cAMP)-cAMP repressor protein (Crp) complex [225]. However, it has been proposed that under these conditions, medium- or short-chain acyl-CoA is the substrate for β-oxidation, not long-chain acyl-CoA [67,225]. These medium and short chains of acyl-CoA also bind FadR and prevent binding to the operator sequence of the *fad* genes, leaving them active during the self-digestion process [225]. Critically, although lipid metabolism and fatty acid oxidation are known to be a critical survival mechanism for cancer persisters by providing energy molecules [28,226,227], this remains largely unexplored for prokaryotic persisters.

## 5. Links between Metabolism and Cellular Self-Digestion

Bacteria must produce a significant amount of energy molecules and building blocks to meet their high metabolic demands. This may lead to metabolic stress that promotes cellular self-digestion. For example, metabolic activity can result in free radical formation via the respiratory chain [228], which can damage proteins, RNAs, DNAs, and lipids, thereby initiating their cellular degradation [229,230].

**Figure 1:**
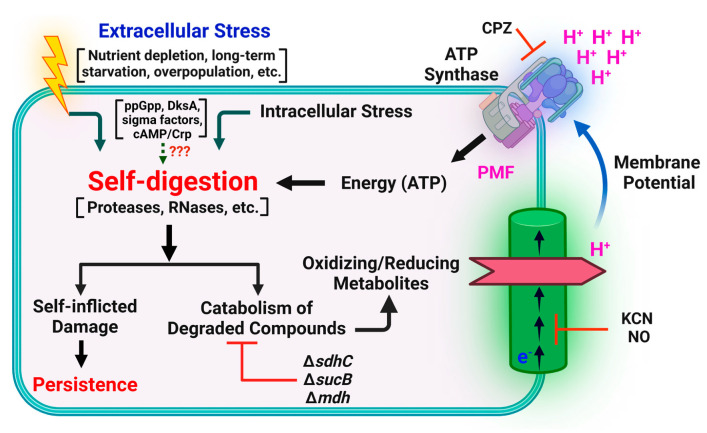
**Self-digestion mediated stationary-phase metabolism in bacteria.** Self-digestion enables cells to transiently tolerate starvation conditions by recycling essential energy molecules. Perturbing the proposed metabolic mechanism genetically (deleting TCA cycle enzymes) [55], chemically (chlorpromazine (CPZ) [232], potassium cyanide (KCN) [55], and nitric oxide (NO) [245] treatments), and environmentally (removing O_2_) [55] can reduce persister formation during the stationary phase.

Although the synthesis of glycolytic enzymes, as well as pyruvate formate lyase, phospho-transacetylase, and acetate kinase, and the subsequent downregulation of enzymes associated with anabolic pathways, has been observed during self-digestion in stationary phase [101,231], persister cells may have unique metabolic mechanisms (Figure 1). In one previous study, we explored the relationship between metabolic activity and persistence using fluorescence-activated cell sorting (FACS) and a redox sensor green dye that measures cytochrome and oxidoreductase rates in the electron transport chain (ETC). With this system, we detected a positive correlation between ETC activity and persistence in stationary-phase cultures [55]. To further determine whether metabolic activity in stationary phase is involved in persister formation, we treated cells with metabolic inhibitors or transferred them to anaerobic conditions in early-stationary phase and measured persister cell levels in late-stationary phase [55,232]. We found that these treatments significantly reduce persister cell levels, confirming an essential role for metabolism in persister cell formation.

Given that the metabolism of non-growing cells primarily derives from the digestion of endogenous cellular components, such as phospholipids, ribosomes, and proteins, during stationary phase [55], we further measured cell size, protein levels, and rRNA integrity in cell cultures with increased (i.e., untreated late-stationary phase) or decreased (i.e., metabolic inhibitor-treated or anaerobic-transferred late-stationary phase) levels of persister cells [55]. We found that untreated late-stationary-phase cells contain significantly more degraded rRNAs and proteins and are markedly smaller than metabolic inhibitor-treated or anaerobically transferred stationary-phase cells [55]. We also determined that deletion of metabolic genes encoding the citric acid (TCA) cycle and ETC enzymes reduces persister levels by preventing digestion of intracellular components, yielding cells that are more vulnerable to cell death when exposed to antibiotics in fresh medium [55,232] (Figure 1).

Although persister cell metabolism is significantly reduced compared to that of exponentially growing cells [13,37,102,103,233], persister cells must still undergo active energy metabolism in order to maintain their adenylate energy charge. Notably, there is evidence that self-digestion mediates the metabolism of persister cells, particularly those formed throughout the stationary phase (Figure 1). In fact, numerous independent studies have shown that persisters can harbor ETC activities [55,234], catabolize certain substrates to generate proton motive force (PMF) [234,235,236], produce energy molecules [237,238], and drive the futile production and degradation of RNA, leading to energy generation and dissipation [239]. Persister cells must also be able to repair antibiotic-induced damage to survive [16,17], and most repair mechanisms (e.g., DNA repair) are strongly ATP-dependent [240,241,242,243]. Further, a number of independent groups have shown that deletion of enzymes associated with the TCA cycle and ETC (e.g., *sdhA, sucA, mdh*) drastically reduce persister levels, indicating the importance of energy metabolism in persister cell formation and/or survival [21,55,244].

Metabolism involves a highly complex enzymatic network that is controlled by a number of transcriptional regulators, including ArcA, Cra, Crp, DksA, Fnr, Lrp, and Rpos, whose expression levels are drastically altered when cells enter stationary phase [140,246,247,248,249,250,251,252]. Critically, these regulators may be involved in self-digestion and in mediating the persister metabolism. Mutants deficient in *arcA,* for example, lose their culturability rapidly after a few days in stationary phase [101]. Cyclic AMP (a product of Cya enzyme) along with its receptor protein, Crp, may also play an important role in bacterial persistence. Persister cells in *E. coli* and *S. aureus* were previously shown to metabolize specific carbon sources and become susceptible to aminoglycoside (AG), which inhibits protein synthesis [234,235,253]. The aminoglycoside potentiation was further extended to Gram-negative pathogens, *Salmonella enterica* and *Klebsiella pneumoniae*, in a subsequent study [236] that also confirms earlier evidence of nascent protein synthesis in persisters [254]. Using the AG-potentiation assay, we found that a panel of carbon sources could not potentiate the AG-mediated killing of persisters derived from Δ*crp* and Δ*cya* strains, indicating a role for these regulators in this process [47]. AG uptake is a unique, energy-requiring process, requiring both the electrochemical potential and the proton gradient across the cytoplasmic membrane [234]. Thus, the fact that persisters can efficiently metabolize certain substrates (e.g., glucose or glycerol) and generate PMF [234,235] supports the existence of active energy metabolism in these cells. However, we note that existence of an active mechanism does not necessarily imply “an upregulation” in that mechanism. The metabolism of persister cells is still likely to be lower than that of exponentially growing cells, which are metabolically highly active [13,37,102,103,233]. Regardless, this does not refute the proposed metabolic model highlighting the reliance of persister cell survival on energy metabolism. In fact, the addiction of some non-proliferating persister types (e.g., cancer persisters) to oxidative phosphorylation has repeatedly been shown [226,227,255,256,257,258].

## 6. Importance of Autophagy in Drug Tolerance

Given the similarities between prokaryotic and eukaryotic persisters [259], it is appealing to draw parallels between bacterial and mammalian persister cells and to expect that knowledge gained from one will enrich our understanding of the other [55]. Although the “persister” term has recently been used in cancer research [28,29,30], drug tolerance mechanisms in tumor cells that are not mediated by heritable mutations have long been known in the field. Indeed, a lot of mechanisms associated with bacterial persisters have been already identified in drug-tolerant cancer cells, such as enhanced efflux activities (e.g., higher rate of drug export from the cell), enhanced repair activities (e.g., efficient repair of drug-induced cell damage), active bypass pathways (e.g., alternative pathways to avoid the drug target), or altered cell metabolism (e.g., a distinct set of active metabolic reactions) [260,261,262].

There are also similarities between cellular self-digestion and autophagy. However, due to the lack of organelles and complex structures in bacteria, a compartmentalized autophagy is not observed in these cells. Autophagy in eukaryotes promotes cell survival and generates energy and intermediate molecules for vital anabolic processes by degrading cellular components in lysosomes [263]. Within these lysosomes, proteins, DNAs, RNAs, polysaccharides, and lipids are hydrolyzed by a diverse range of degradative enzymes [264]. Lysozymes contain approximately 50 different hydrolytic enzymes [265], including the Lon protease [266], which was first identified in bacteria and is known to be a crucial persistence molecule, as discussed above.

Depending on the mechanism that mediates transport of cytosolic cargo to lysosomes, eukaryotic autophagy can be categorized into three major types: macroautophagy, microautophagy, and chaperone-mediated autophagy (CMA) [267]. Although, macroautophagy and microautophagy are similar in terms of initiation, termination, and capacity for the sequestration of large structures, they are distinct pathways [268]. The formation of autophagosomes is a hallmark of macroautophagy and consists of several distinct steps (i.e., nucleation, elongation, and closure of the double-membraned vesicle) [269]; these are mediated by a cluster of proteins known as autophagy-related proteins (ATGs). Once the autophagosome is formed, it fuses with a lysosome, and a single-membrane vesicle (i.e., the autophagic or Cvt body) is released into the lumen. In contrast, during microautophagy, cargos are directly engulfed and taken up by the lysosomal membrane as result of local deformation and rearrangement of the membrane, allowing the cytosolic cargos to be degraded by vacuolar hydrolases and enzymes within the lysosome [264]. The resulting macromolecules such as amino acids or fatty acids from the lysosomal degradation are recycled back into the cytosol via membrane permeases to be used in anabolic processes. In CMA, the degradation of soluble cytosolic proteins in lysosomes is highly selective. Substrate selection in CMA is regulated by cytosolic chaperones that recognize pentapeptide motifs in the amino acid sequence of the substrate proteins [270]. In this type of autophagic process, substrates are not engulfed, but, instead, are translocated across the lysosomal membrane in a receptor-mediated manner [271].

In eukaryotes, the autophagy regulatory network is highly complex and tightly connected to redundant signaling pathways, some of which are related to the cell cycle and proliferation, including the mammalian target of rapamycin kinase (TOR), nuclear factor of kappa light polypeptide gene enhancer (NFKB/NF-κB), mitogen-activated protein kinase (MAPK), and tumor protein p53 (TP53) cascades [272]. Notably, autophagy may play a critical role in cancer persistence, as there is evidence for reciprocal interactions between autophagy and cell cycle arrest, the hallmark of cancer persistence [273]. Cancer persisters can escape cell death pathways (e.g., apoptosis) by inactivating their cell-proliferation signaling pathways during treatment. In fact, many targeted therapeutics may induce cell dormancy by directly inhibiting cell-proliferation signaling pathways, whereas chemotherapeutics may indirectly stimulate growth arrest by activating stress signaling pathways [274,275,276,277]. Critically, the mechanisms associated with cell growth arrest may be mediated by the same signaling pathways that are involved in autophagy [273].

The degree of stress (e.g., starvation) that a cell is under can determine if autophagy will be used as a means for survival [263] or programmed cell death [278]. However, autophagy is not only a defense mechanism for starvation, but a necessary function for molecular recycling and maintaining the homeostasis of non-starved cells [263]. Knockout of ATGs has been shown to cause severe developmental problems in mice, including abnormalities at the cellular level [279], obesity [280], lung dysfunction [281], tumorigenesis [282,283], and death [283]. Critically, dependence of tumor cell survival and growth on basal autophagy has also been demonstrated via ATG knockout [284], and numerous observations support a key role for autophagy in cancer growth and survival. In one instance, it was shown that deletion of ATG7 leads to metabolic and proliferative problems in cancer, causing cancer cells to become more sensitive to starvation and more dependent on glutamine [285]. RAS-mutated cancers were also found to exhibit upregulated autophagy, leading to sustained TCA cycle metabolism [286], increased levels of glycolysis [287], and enhanced tumorigenesis [286,288].

In addition to providing support for cancer growth and survival under stress, autophagy can help cancer cells resist treatment. Increasing levels of autophagic flux have been correlated with higher cancer cell survival rates and shortened patient survival times in melanoma [289]. The specific mechanisms that mediate this phenomenon have not been fully elucidated; however, autophagy appears to play various protective roles in cancer, depending on the cancer type and the treatment method. For example, in BRAF-mutated cancers, BRAF inhibition leads to endoplasmic reticulum (ER) stress, which subsequently increases autophagic activity, protecting the cells from apoptosis and maintaining mitochondrial activity [290]. Autophagy also makes mTOR-mutated cancer cells tolerant to mTOR inhibitors by eliminating receptor-interacting protein kinases (RIPKs), which promote necroptosis when autophagy is inhibited [291]. Overall, both autophagy and self-digestion seem to be evolutionarily conserved mechanisms that allow organisms to survive undesirable environmental conditions. Both processes can be activated in response to extracellular stress conditions such as nutrient deprivation; maintain cellular energy homeostasis; may use similar degradative enzymes (such as Lon); and can lead to growth arrest, and therefore tolerance to both antibiotics (for bacteria) and chemotherapeutics (for cancer cells).

## 7. Concluding Remarks

Persistence and self-digestion (or autophagy), which appear to be evolutionarily conserved phenomena, are observed in many prokaryotic and eukaryotic cell types. These processes allow organisms to survive in undesirable environmental conditions, leading to formation of persister cells. Critically, mapping a self-digestion-mediated persistence mechanism from its initial exogenous stress trigger, through its signal transduction, to the source of antibiotic tolerance, may provide us an opportunity to categorize previously identified mechanisms within one complex network. Such a strategy may also uncover novel antipersister therapeutic approaches, as inhibition of intracellular degradation is known to reduce persister formation [55,234,245]. Conversely, stimulating self-digestion might also be useful as an alternative antipersister strategy, due to the fact that enhanced intracellular degradation can also be detrimental to persister cells [27,54]. However, the study of cellular self-digestion mechanisms is challenging, and a number of critical questions remain to be addressed, some of which are as follows:

(i) If a proposed mechanism is essential for persister formation and survival, genetically perturbing the mechanism should eliminate persisters or reduce their levels; however, this method may not be ideal for redundant systems. Self-digestion-mediated persistence is potentially a collective effect of many different degradative enzymes, which makes it difficult to test using conventional methods. One way to investigate these mechanisms is to perform single-cell analysis. With the use of antibiotic treatments and fluorescent reporters for degradative enzymes, a correlation between persistence and the enzymes expression levels can be performed in cell populations where self-digestion is significantly upregulated (e.g., late-stationary-phase cultures).
(ii) Although Kim et al. claim that persisters and “viable but non-culturable” (VBNC) cells represent the same phenotypes [54], the VBNC state is thought to be a transitory phase on the spectrum between persistence and cell death [292,293]. While persister cells can exit from persistence state (stochastically or deterministically) and colonize, the resuscitation of VBNC cells is rarely observed [35,235,293,294]. In fact, bacteria associated with asymptomatic infections may be in a non-replicating or slowly replicating state and cannot be easily cultured in vitro [295,296,297], and this “viable but non-culturable” state observed in pathogenic bacteria has long been known [296]. Further, a number of independent groups have shown antibiotic-treated cultures contain many more VBNC cells than persisters [35,37,235,298]. If persisters and VBNC cells represent two distinct phenotypes on the live- and dead-cell spectrum, then, a threshold level of intracellular degradation may play a critical role in the phenotypic switch between persistence and the VBNC state, which remains to be validated.
(iii) Persister metabolism is a controversial topic, reflecting the complexity and diversity of persister cell formation, survival, and resuscitation mechanisms, as well as the influence of culture conditions [31,61,299]. Although persisters are mostly non-growing cells [33,300,301,302], and their metabolism is generally lower than that of exponentially growing cells [13,37,102,103,233], these phenotypes might be at a metabolic steady state, providing energy molecules necessary for their survival [237,239]. Although it is well established that autophagy plays a crucial role in the metabolism of drug-tolerant cancer cells, it remains to be determined whether this is also true for bacterial persisters.
(iv) The levels of global regulators, such as Rpos, ppGpp, and cAMP/Crp, are significantly altered in cells during their transition to stationary phase [110,116,117,126,144,147]. However, we still do not know if these molecules regulate expression of degradative enzymes, as the promoters of many genes encoding degradative enzymes are not well characterized [303]. Constitutive expression of degradative enzymes may result in their accumulation in stationary phase, which would make intracellular degradation more apparent in stationary-phase cells, where cell growth and protein synthesis are minimal. However, this has yet to be validated.
(v) Recently, several groups have uncovered a correlation between protein aggregation and bacterial persistence, although protein aggregation seems to be associated with the VBNC phenotype [12,304,305,306,307]. While these results may contradict with self-digestion-mediated persister mechanisms at first glance, it is well known that protein aggregation can induce autophagy in mammalian cells [308,309,310]. Whether a similar phenomenon is also present in bacterial cells is yet to be determined.

## Data Availability

Not applicable.
